# Comparison of ischemic stroke diagnosis models based on machine learning

**DOI:** 10.3389/fneur.2022.1014346

**Published:** 2022-12-05

**Authors:** Wan-Xia Yang, Fang-Fang Wang, Yun-Yan Pan, Jian-Qin Xie, Ming-Hua Lu, Chong-Ge You

**Affiliations:** ^1^Laboratory Medicine Center, Lanzhou University Second Hospital, Lanzhou, China; ^2^Anesthesiology Department, Lanzhou University Second Hospital, Lanzhou, China

**Keywords:** ischemic stroke, machine learning, artificial neural network, diagnostic model, transcriptomics

## Abstract

**Background:**

The incidence, prevalence, and mortality of ischemic stroke (IS) continue to rise, resulting in a serious global disease burden. The prediction models have a great value in the early prediction and diagnosis of IS.

**Methods:**

The R software was used to screen the differentially expressed genes (DEGs) of IS and control samples in the datasets GSE16561, GSE58294, and GSE37587 and analyze DEGs for enrichment analysis. The feature genes of IS were obtained by several machine learning algorithms, including the least absolute shrinkage and selector operation (LASSO) logistic regression, the support vector machine-recursive feature elimination (SVM-RFE), and the Random Forest (RF). The IS diagnostic models were constructed based on transcriptomics by machine learning and artificial neural network (ANN).

**Results:**

A total of 69 DEGs, mainly involved in immune and inflammatory responses, were identified. The pathways enriched in the IS group were complement and coagulation cascades, lysosome, PPAR signaling pathway, regulation of autophagy, and toll-like receptor signaling pathway. The feature genes selected by LASSO, SVM-RFE, and RF were 17, 10, and 12, respectively. The area under the curve (AUC) of the LASSO model in the training dataset, GSE22255, and GSE195442 was 0.969, 0.890, and 1.000. The AUC of the SVM-RFE model was 0.957, 0.805, and 1.000, respectively. The AUC of the RF model was 0.947, 0.935, and 1.000, respectively. The models have good sensitivity, specificity, and accuracy. The AUC of the LASSO+ANN, SVM-RFE+ANN, and RF+ANN models was 1.000, 0.995, and 0.997, respectively, in the training dataset. However, the AUC of LASSO+ANN, SVM-RFE+ANN, and RF+ANN models was 0.688, 0.605, and 0.619, respectively, in the GSE22255 dataset. The AUC of the LASSO+ANN and RF+ANN models was 0.740 and 0.630, respectively, in the GSE195442 dataset. In the training dataset, the sensitivity, specificity, and accuracy of the LASSO+ANN model were 1.000, 1.000, and 1.000, respectively; of the SVM-RFE+ANN model were 0.946, 0.982, and 0.964, respectively; and of the RF+ANN model were 0.964, 1.000, and 0.982, respectively. In the test datasets, the sensitivity was very satisfactory; however, the specificity and accuracy were not good.

**Conclusion:**

The LASSO, SVM-RFE, and RF models have good prediction abilities. However, the ANN model is efficient at classifying positive samples and is unsuitable at classifying negative samples.

## Introduction

The Global Burden of Diseases, Injuries, and Risk Factors Study (GBD) showed that there were 12.2 million incident cases of stroke, 101 million prevalent cases of stroke, and 6.55 million deaths from stroke in 2019 (1). Globally, the incidence and mortality of stroke are on the rise, and stroke remains the second leading cause of death (2). Especially in China, cerebrovascular disease is the first cause of death, and the lifetime risk of stroke in the Chinese population ranks first in the world (3). In 2019, there were 3.94 million new stroke cases, 2.19 million deaths from stroke, and 28.76 million prevalent cases of stroke, of which ischemic stroke (IS) accounted for 84.1% in China (4).

The etiology and pathogenesis of IS are not fully understood. According to epidemiological investigations, IS may be associated with hypertension, high BMI, hyperglycemia, environmental particulate matter pollution, and smoking (1, 5). As modern medicine tends to be individualized, prevention and treatment strategies based on patient genetic information have always been ideal treatment methods for medical practitioners. Studies (6) have found that genetic factors also play a very important role in the occurrence of IS. At present, more and more studies believe that the occurrence and poor prognosis of IS are related to the abnormal expression of genes (7). However, multiple genes are often involved in the occurrence of IS. This inspired us to explore diagnostic and prognostic methods for IS by using multiple disease-specific genes.

At present, there are some limitations to the IS diagnostic techniques commonly used in clinical practice. The diagnosis of IS mainly relies on typical clinical symptoms and brain imaging (8), while approximately 50% of early IS diagnoses lack specificity in imaging (9). In addition, most patients are irreversible by the time the diagnosis is confirmed, resulting in a poor prognosis. Although scholars have done a great deal of work in finding biomarkers for IS diagnosis or prognosis, few biomarkers are available in clinical practice (10). Existing predictive models are mostly based on demographic data and clinical parameters, which may have a high risk of bias and fail to make reliable clinical decisions (11). Machine learning research is developing rapidly and has become one of the important topics in the field of artificial intelligence. At present, machine learning has become a research hotspot in the field of medical and health data mining (12). Machine learning algorithms such as the least absolute shrinkage and selector operation (LASSO), support vector machine-recursive feature elimination (SVM-RFE), Random Forest (RF), and the neural network have been proven to be of great value in diagnosing stroke (13–15).

In this study, we screened differentially expressed genes (DEGs) between IS and control samples in the Gene Expression Omnibus (GEO) database; used LASSO, SVM-RFE, and RF to screen out IS feature genes; and constructed a disease diagnosis model of IS to evaluate the performance of different models on predicting IS.

## Methods

### Microarray data and processing

The expression profile data and corresponding platform annotation information of microarray datasets, such as GSE16561, GSE58294, GSE37587, GSE22255, and GSE195442, were downloaded from the GEO database (https://www.ncbi.nlm.nih.gov/geo/). GSE16561, GSE58294, and GSE37587 were integrated as training datasets, and GSE22255 and GSE195442 were used as test datasets, as shown in Table 1. The R software (version 4.1.0) was used to transform the probe names of GSE16561, GSE58294, GSE37587, GSE22255, and GSE195442 matrix data into gene names. After the integration of the GSE16561, GSE58294, and GSE37587 datasets, the data were normalized by log2 transformation for data with large values and averaging for repeated probes. The “sva” package was used to calibrate batch effects. The principal component analysis (PCA) diagram before and after calibration was drawn using the ggplot2 package. Since there are 47 control samples and 176 IS samples in the integrated training dataset, there is a class imbalance. We used the SMOTE algorithm (16) to adjust for class imbalance. The R software “UBL” package was used.

**Table 1:** Ischemic stroke datasets from the GEO database.

**Group**	**Dataset**	**Reference**	**Data type**	**Platform**	**Stroke**	**Control**
Training dataset	GSE16561	Barr (17)	Microarray	GPL6883	39	24
Training dataset	GSE58294	Stamova (18)	Microarray	GPL570	69	23
Training dataset	GSE37587	Barr (19)	Microarray	GPL6883	68	0
Test dataset	GSE22255	Krug (20)	Microarray	GPL570	20	20
Test dataset	GSE195442	Yang (21)	Microarray	GPL31275	10	10

### Screening for differentially expressed genes (DEGs)

The “limma” package was used to screen DEGs of the integrative data of GSE16561, GSE58294, and GSE37587. The screening criteria were set as |log_2_FC| > 0.6 and the adjusted *P-*value was <0.05. The heatmap and volcano plot of DEGs were drawn using the “pheatmap” and “ggplot2” packages, respectively.

### Enrichment analysis

To understand the functions of DEGs, we used the R software “clusterProfiler” package to conduct a Gene Ontology (GO) enrichment analysis and a Gene Set Enrichment Analysis (GSEA) on DEGs. An adjusted *P*-value of <0.05 was considered statistically significant. GO enrichment analysis includes a biological process (BP), a cellular component (CC), and a molecular function (MF).

### Feature selection and model evaluation

To screen out the feature genes of IS, the R was used to perform machine learning analysis on DEGs. The “glmnet” package was used to construct the LASSO model with penalty parameter tuning conducted by ten-fold cross-validation. The response type was set as binomial, and the alpha was set as 1. We selected the feature genes with the minimum error. Besides, the “e1071” package was used to establish the SVM-RFE model to screen out the genes with the minimum cross-validation error. *k* = 10 was the setting for the *k*-fold cross-validation, and the parameter of halving above was identified as 50. The “randomForest” package was used to establish the RF model. The RF model was established to find out the number of random forest trees with the minimum error. We selected 272 trees as the parameter of the random forest model. The “pROC” software package was used to draw the receiver operating characteristic (ROC) curve to validate the accuracy of the model. The dimensionality importance value of the RF model was obtained using the decreasing accuracy method (Gini coefficient method). The performance of prediction models generated by machine learning classifiers was assessed using classification sensitivity, specificity, and the area under the curve (AUC).

### Construction and validation of the ANN model

To build and evaluate the performance of the artificial neural network (ANN) model, we performed gene scoring for feature genes, and the scoring rule was set as follows: if the expression of upregulated genes was greater than the median value, the score was 1; otherwise, the score was 0. If the expression of downregulated genes was greater than the median value, the score was 0; otherwise, the score was 1. The R software package “neuralnet” was used to construct the ANN model of feature genes according to the gene score. We set the hidden layer of the LASSO+ANN, SVM-RFE+ANN, and RF+ANN models as 1. The number of neurons in the hidden layers of the LASSO+ANN, SVM-RFE+ANN, and RF+ANN models was set as 8, 5, and 6, respectively. The activation function “logistic” was used. The IS disease classification model was constructed using the obtained gene weight information.

## Results

### Batch calibration and SMOTE algorithm

**Figure 1:**
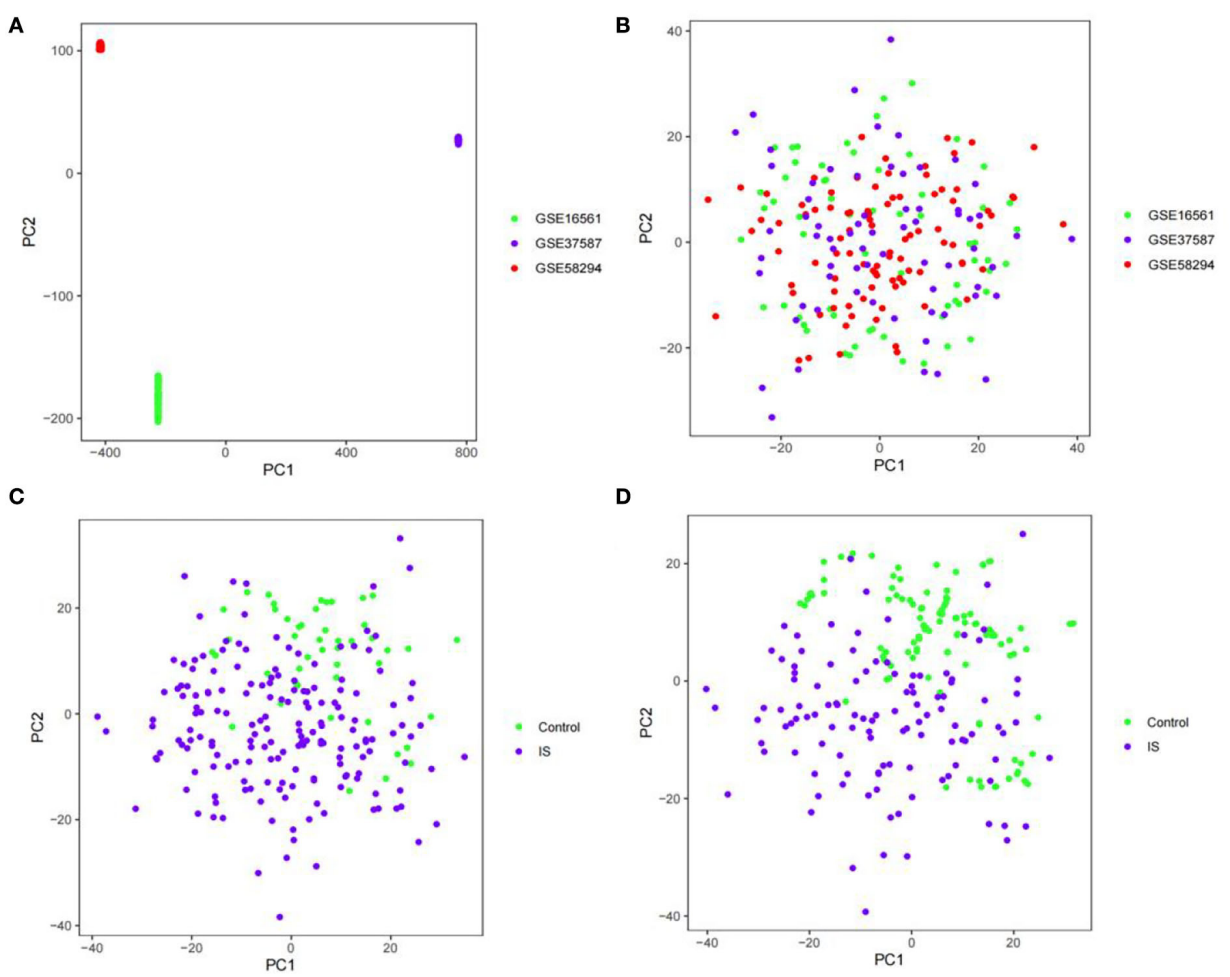
PCA diagram. **(A)** PCA diagram of GSE16561, GSE58294, and GSE37587 datasets before calibration. **(B)** PCA diagram of GSE16561, GSE58294, and GSE37587 datasets after calibration. **(C)** PCA diagram of class distribution before SMOTE. **(D)** PCA diagram of class distribution after SMOTE.

The GSE16561, GSE58294, and GSE37587 datasets were integrated. To reduce the differences between batches, batch calibration was performed on the two datasets, and PCA was used to verify the effect of data calibration (Figures 1A,B). The class distribution in the integrated dataset is not equal, which is prone to class imbalance. Training classification algorithms with imbalanced data provide inefficient prediction models, which may perform poor classification on a smaller number of samples. Hence, we used SMOTE to fix class imbalance (Figures 1C,D).

### Differential gene analysis

**Figure 2:**
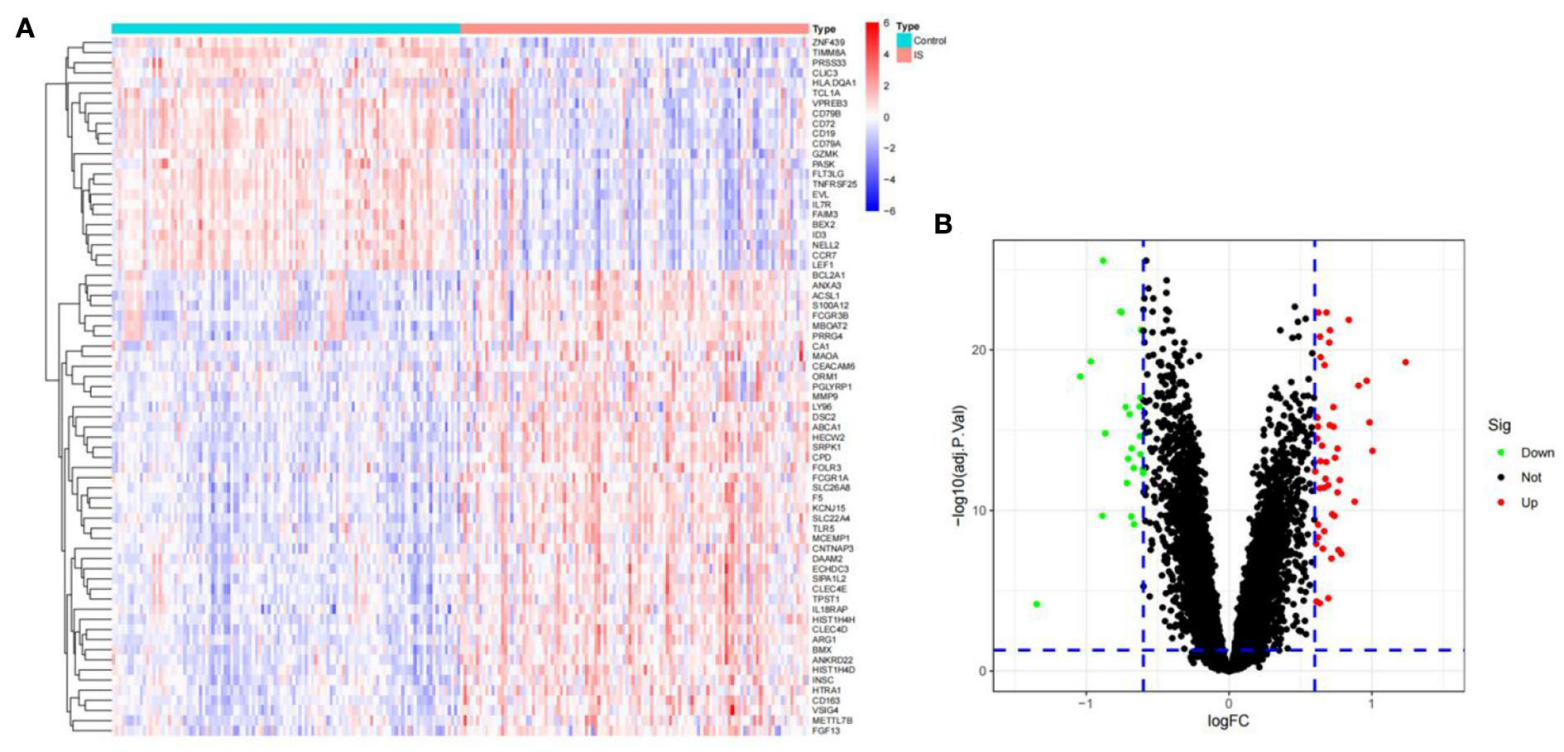
The DEGs between ischemic stroke and control group in the GSE16561, GSE58294, and GSE37587 datasets. **(A)** Heatmap of DEGs. The red and blue represent the significantly upregulated and downregulated DEGs. **(B)** Volcano plot of DEGs. These genes consist of 46 upregulated genes and 23 downregulated genes. The screening criteria were set as |log_2_FC|> 0.6 and adjusted *P*- value of < 0.05.

To identify the DEGs from IS and control samples, we conducted a Bayesian test on the training dataset and obtained a total of 69 DEGs, of which 46 were upregulated and 23 were downregulated (Figures 2A,B).

### Function and pathway enrichment analysis

**Figure 3:**
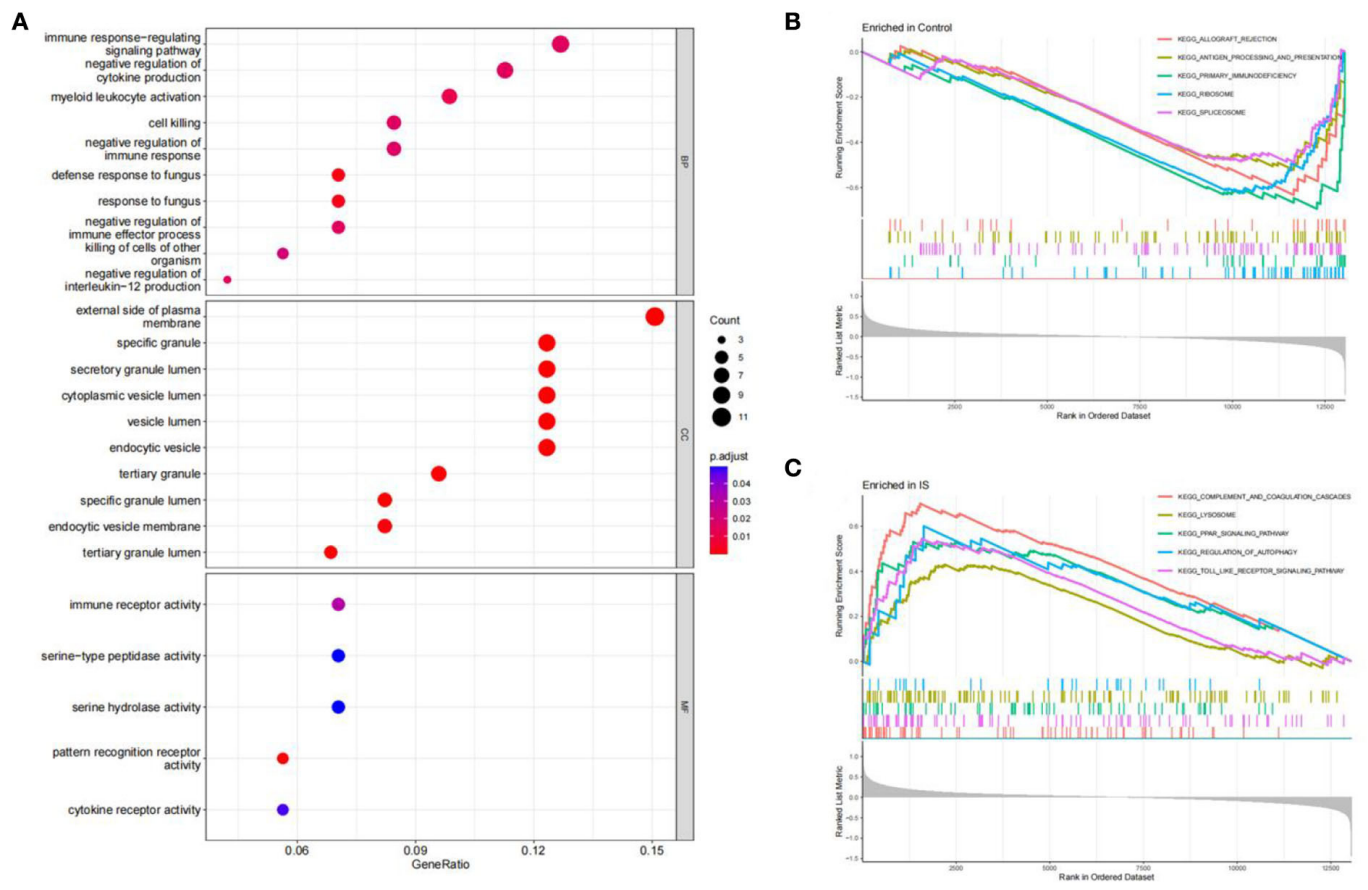
Function enrichment analysis. **(A)** GO enrichment analysis of DEGs. The size of the circle indicates the number of genes. The screening criterion was set as adjusted *P* < 0.05. **(B,C)** Enrichment plots from GSEA analysis in the control group and IS group.

The R software was used to perform enrichment analysis on 69 DEGs, as shown in Figure 3. DEGs were mainly enriched in the immune response and the inflammatory response. The biological process involved immune response-regulating signaling, negative regulation of cytokine production, and negative regulation of immune response. The cellular component mainly focused on some granule lumens and granule membranes. The molecular function analysis showed that most of the genes were involved in immune receptor activity, serine-type peptidase activity, serine hydrolase activity, pattern recognition receptor activity, and cytokine receptor activity (Figure 3A).

The GSEA analysis indicated that the most enriched pathways in the control group were allograft rejection, antigen processing and presentation, primary immunodeficiency, ribosome, and spliceosome (Figure 3B). In contrast, complement and coagulation cascades, lysosome, PPAR signaling pathway, regulation of autophagy, and toll-like receptor (TLR) signaling pathway were enriched in the IS group (Figure 3C).

### Screening for feature genes *via* machine learning

**Figure 4:**
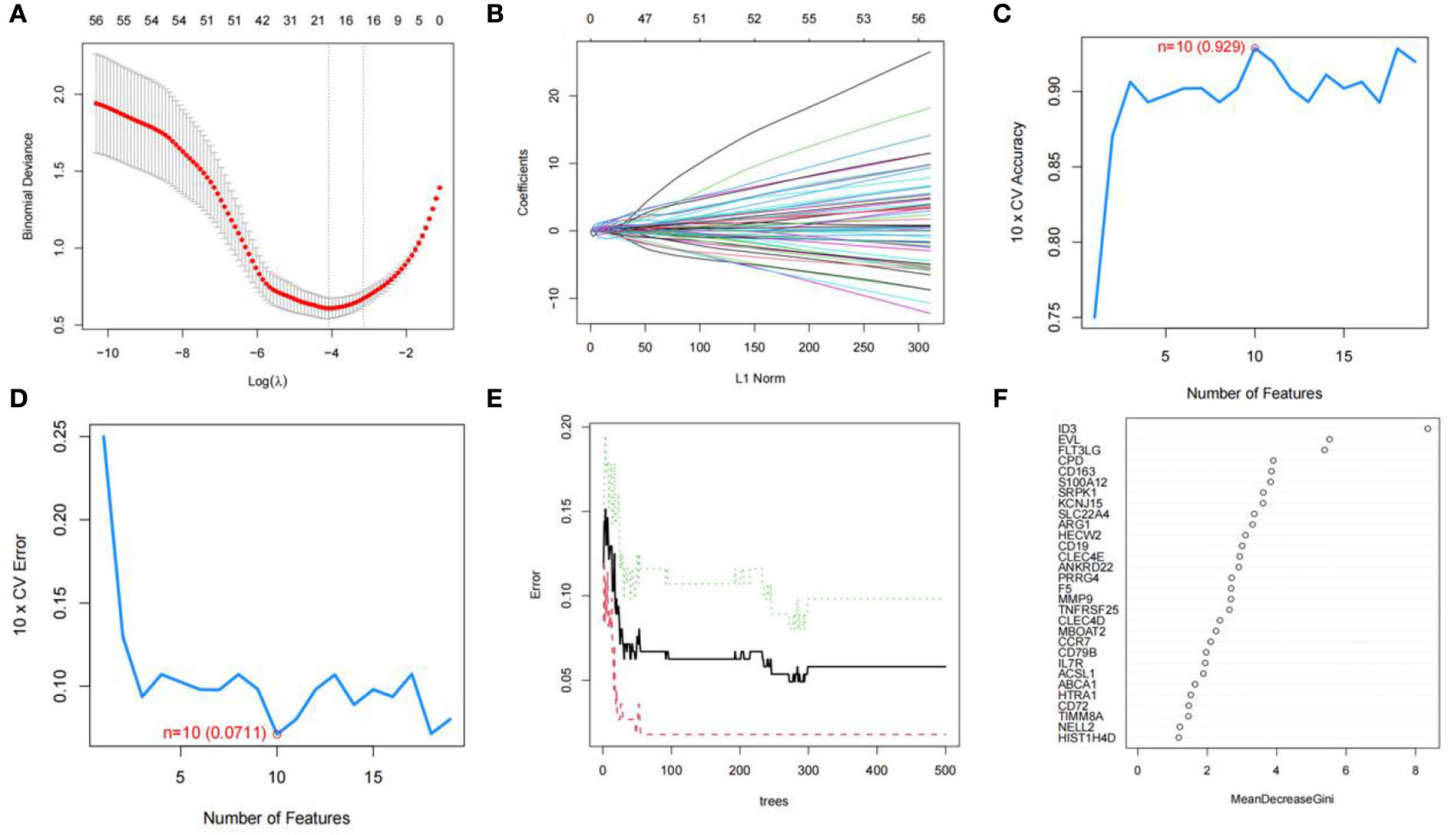
Screening for feature genes. **(A)** Identification of the optimal penalization coefficient lambda (λ) in the LASSO model. **(B)** Cross-validation for tuning parameter selection in the LASSO model. **(C,D)** A plot of genes selection *via* SVM-RFE algorithm. **(E)** The influence of the number of decision trees on the error rate. The *x-*axis represents the number of decision trees, and the *y*-axis indicates the error rate. **(F)** Results of the Gini coefficient method in RF model. The *x*-axis indicates the genetic variable, and the *y*-axis represents the importance index.

We used R software to perform machine learning analysis on 69 DEGs. The feature genes selected by LASSO (Figures 4A,B) and SVM-RFE (Figures 4C,D) were 17 and 10, respectively. The number of random forest trees with the minimum error of the RF model was 272 (Figure 4E). The 12 genes with an importance value >3 were selected as disease-specific genes (Figure 4F). The feature genes screened by the algorithms are shown in Table 2.

**Table 2:** Feature genes screened by machine learning algorithms.

**Algorithms**	**Genes**
LASSO	CPD, CLEC4D, CD163, CD19, ANKRD22, CD79B, HIST1H4D, HIST1H4H, TIMM8A, CLIC3, HTRA1, MAOA, LY96, PRSS33, FCGR3B, METTL7B, FOLR3
SVM-RFE	CLEC4D, ZNF439, PGLYRP1, HECW2, FAIM3, ANKRD22, CD79A, EVL, LY96, CD72
RF	ID3, EVL, FLT3LG, CPD, CD163, S100A12, SRPK1, KCNJ15, SLC22A4, ARG1, HECW2, CD19

### Effectiveness of machine learning models

**Figure 5:**
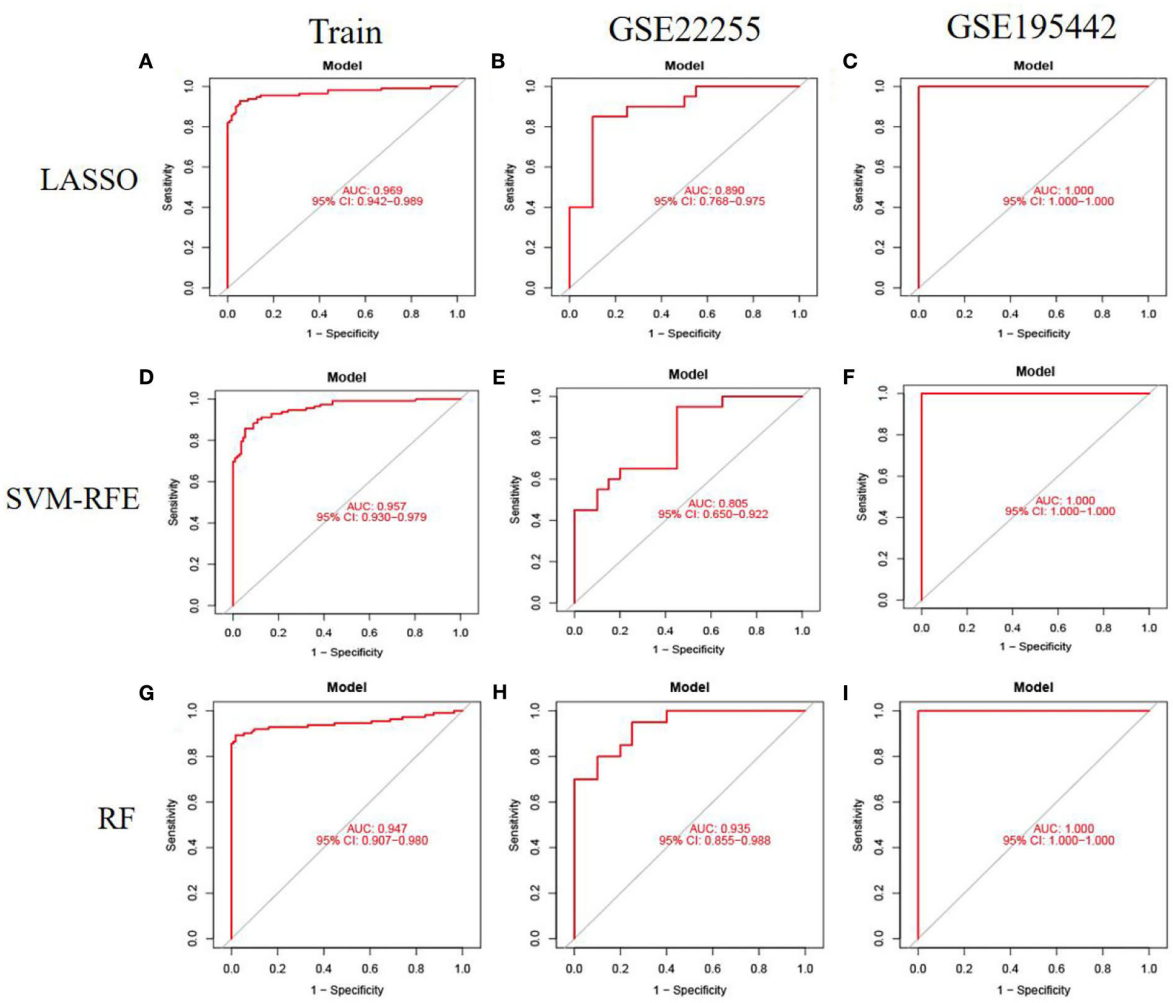
Model accuracy evaluation. **(A–C)** The ROC curves for using LASSO to estimate accuracy in training, GSE22255, and GSE195442 datasets. **(D–F)** The ROC curves for using SVM-RFE to estimate accuracy in training, GSE22255, and GSE195442 datasets. **(G–I)** The ROC curves for using RF to estimate accuracy in training, GSE22255, and GSE195442 datasets.

To evaluate the prediction performance of the machine learning model, we first constructed the model by LASSO, SVM-RFE, and RF. In the training dataset and GSE22255 and GSE195442 test datasets, the AUC of the LASSO model was 0.969, 0.890, and 1.000, respectively (Figures 5A–C); the AUC of the SVM-RFE model was 0.957, 0.805, 1.000 (Figures 5D–F), respectively, and the AUC of the RF model was 0.947, 0.935, 1.000 (Figures 5G–I), respectively. In addition, the models have good sensitivity and specificity (Table 3).

**Table 3:** Comparison of ischemic stroke diagnosis models based on machine learning.

**Models**	**Datasets**	**AUC**	**Sensitivity**	**Specificity**	**Accuracy**
LASSO	Train	0.969 (0.942–0.989)	0.929	0.946	0.938
	GSE22255	0.890 (0.768–0.975)	0.850	0.850	0.850
	GSE195442	1.000 (1.000–1.000)	1.000	1.000	1.000
SVM-RFE	Train	0.957 (0.930–0.979)	0.857	0.946	0.902
	GSE22255	0.805 (0.650–0.922)	0.950	0.550	0.750
	GSE195442	1.000 (1.000–1.000)	1.000	1.000	1.000
RF	Train	0.947 (0.907–0.980)	0.893	0.982	0.938
	GSE22255	0.935 (0.855–0.988)	0.817	0.883	0.850
	GSE195442	1.000 (1.000–1.000)	1.000	1.000	1.000
LASSO+SVM-RFE	Train	0.898 (0.853–0.934)	0.777	0.866	0.822
	GSE22255	0.692 (0.522–0.840)	0.617	0.683	0.650
	GSE195442	0.920 (0.730–1.000)	1.000	0.900	0.950
SVM-RFE+RF	Train	0.899 (0.854–0.939)	0.777	0.973	0.875
	GSE22255	0.647 (0.473–0.820)	0.850	0.500	0.675
	GSE195442	0.850 (0.640–1.000)	0.800	0.900	0.850
LASSO+ANN	Train	1.000 (0.999–1.000)	1.000	1.000	1.000
	GSE22255	0.688 (0.510–0.845)	0.850	0.500	0.675
	GSE195442	0.740 (0.490–0.950)	0.800	0.500	0.650
SVM-RFE+ANN	Train	0.995 (0.988–0.999)	0.946	0.982	0.964
	GSE22255	0.605 (0.420–0.771)	0.700	0.400	0.550
RF+ANN	Train	0.997 (0.991–1.000)	0.964	1.000	0.982
	GSE22255	0.619 (0.429–0.787)	0.750	0.450	0.600
	GSE195442	0.630 (0.360–0.860)	0.700	0.400	0.550

To further evaluate the prediction performance of the combination of machine learning algorithms, we constructed and validated the LASSO+SVM-RFE and SVM-RFE+RF models. The AUC, sensitivity, and specificity of the LASSO+SVM-RFE and SVM-RFE+RF models were also satisfactory, as shown in Table 3.

### Construction and validation of the ANN model

**Figure 6:**
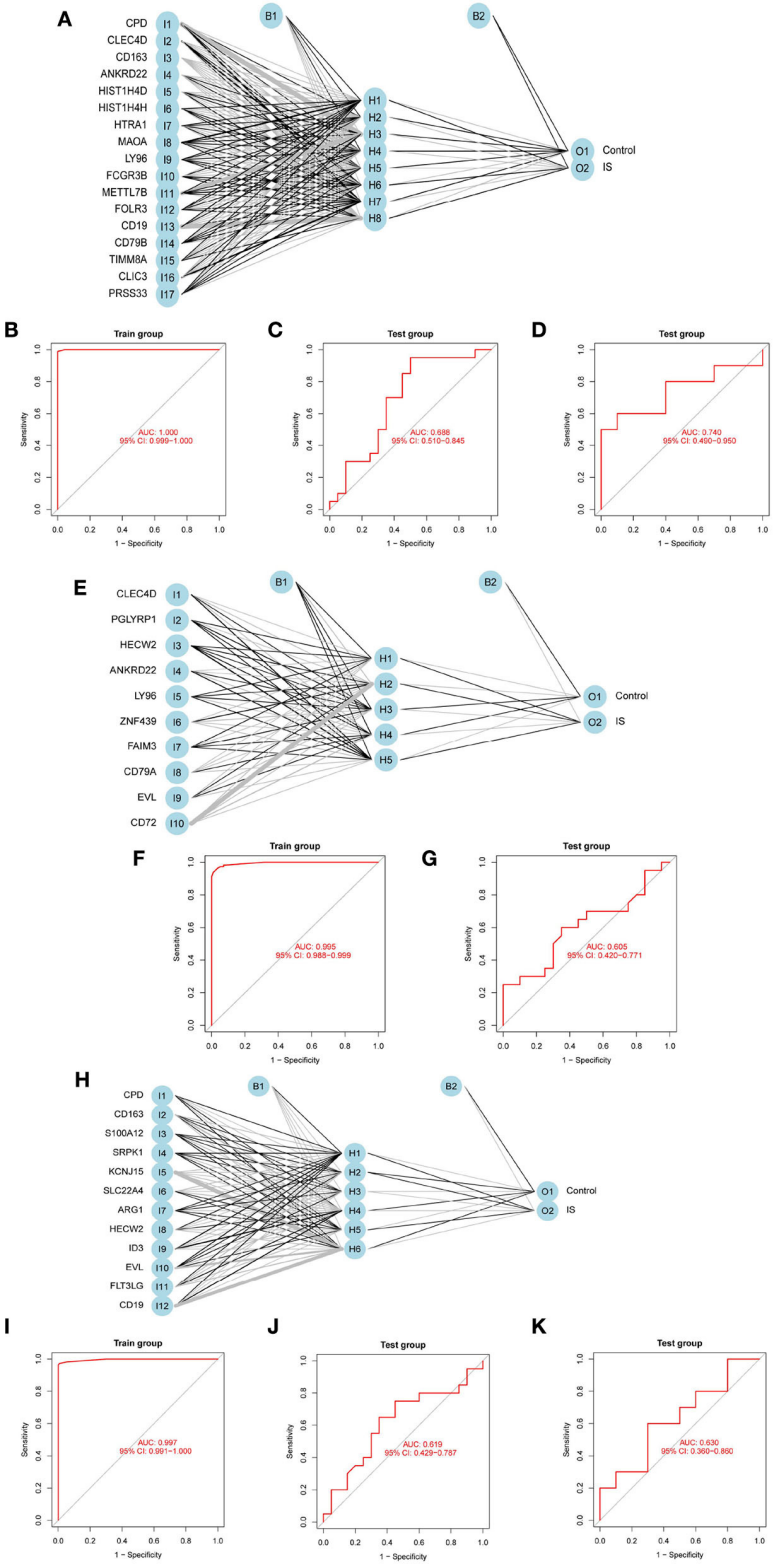
Development and validation of ANN models. **(A,E,H)** Visualization of the LASSO+ANN, SVM-RFE+ANN, and RF+ANN models. **(B–D)** ROC analysis for model prediction of the LASSO+ANN in the training, GSE22255, and GSE195442 datasets. **(F,G)** ROC analysis for model prediction of the SVM-RFE+ANN in the training, GSE22255, and GSE195442 datasets. **(I–K)** ROC analysis for model prediction of the RF+ANN model in the training, GSE22255, and GSE195442 datasets.

To evaluate the prediction performance of the ANN model, we constructed and validated ANN models for feature genes screened by LASSO, SVM-RFE, and RF, respectively. The visualization of the LASSO+ANN, SVM-RFE+ANN, and RF+ANN models is shown in Figures 6A,E,H. The AUC of LASSO+ANN, SVM-RFE+ANN, and RF+ANN models in the training dataset was 1.000, 0.995, and 0.997, respectively (Figures 6B,F,I). The AUC of LASSO+ANN, SVM-RFE+ANN, and RF+ANN in the GSE22255 dataset was 0.688, 0.605, and 0.619, respectively (Figures 6C,G,J). The AUC of LASSO+ANN and RF+ANN in the GSE195442 dataset was 0.740 and 0.630, respectively (Figures 6D,K).

In the training dataset, the sensitivity, specificity, and accuracy of the LASSO+ANN model were 1.000, 1.000, and 1.000, respectively; of the SVM-RFE+ANN model were 0.946, 0.982, and 0.964, respectively; and of the RF+ANN model were 0.964, 1.000, and 0.982, respectively. In the test datasets, the sensitivity (true positive rate) was very satisfactory; however, the specificity (true negative rate) and accuracy were not good. This shows that the ANN model is very efficient at classifying positive samples and is unsuitable at classifying negative samples (Table 3).

## Discussion

In this study, the 69 DEGs identified were mainly involved in the immune response and inflammatory response. Inflammation is one of the initial responses of the immune system to a stimulus. Studies have shown that the immune system plays a very important role in the acute and chronic stages of ischemic damage and in the long-term sequelae of stroke (22). The pathways enriched in the IS group were complement and coagulation cascades, lysosome, PPAR signaling pathway, regulation of autophagy, and TLR signaling pathway. A sudden interruption of IS blood flow can lead to vascular endothelial changes, local retention of blood cells, platelet-leukocyte adhesion, and activation of the coagulation cascade, whereas thrombin induces the expression of adhesion molecules on endothelial cells, disrupts endothelial barrier function, and activates complement C3 and C5 (23). TLR, as part of the innate immune system, plays an important role in the immune response of IS (24). After the occurrence of hypoxic-ischemic events, part of the TLRs present in the endothelial cell membranes is involved in endothelial dysfunction and plays an indispensable role in the activation of inflammatory cascades (25). The autophagy-lysosomal pathway participates in the clearance of aberrant cellular components to maintain protein homeostasis and normal cellular function. Evidence indicated that the impairment of this pathway during cerebral ischemia led to ischemia-induced neuronal necrosis and apoptosis (26).

Stroke is the second leading cause of disability and death worldwide. Currently, there are no effective treatments to improve stroke survival and quality of life. Early diagnosis and intervention of IS play an essential role in reducing deaths.

A great deal of effort has been put into post-IS management, and there are many methods that play a role in assessing unfavorable post-IS outcomes, such as real-time biosignaling (27), quantitative electroencephalography (qEEG) (28), and electromyography (29). Noninvasive qEEG has good discriminative power in the quantitative evaluation of neurological outcomes after stroke compared with known demographic, clinical, and radiographic prognostic markers. Electromyography (EMG) is also considered a potential predictive tool for post-stroke gait and rehabilitation management because it is sensitive to neuromuscular changes induced by IS. Myoelectric biomarkers will help detect gait changes in stroke-impaired patients and determine post-stroke rehabilitation. There are also many methods that can assist in the diagnosis of IS. The imaging biomarker of carotid plaque can also be used to predict stroke risk (30). To date, most studies examining stroke have used MRI or CT images, which can be difficult to diagnose in advance. Studies have found that electrocardiography (31) and echocardiography (32) can also predict IS risk. Although electrocardiography and echocardiography are noninvasive and low-cost diagnostic methods, their low sensitivity can easily lead to misdiagnosis. Therefore, it is necessary to develop a highly sensitive and accurate method for the early diagnosis of IS.

This study aimed to construct prediction models of IS based on transcriptomics using machine learning methods. Overall, among the eight models, the LASSO, SVM-RFE, and RF performed best with the highest values in performance (AUC, sensitivity, specificity) in the training dataset and test datasets, followed by LASSO+SVM-RFE and SVM-RFE+RF, the LASSO+ANN, SVM-RFE+ANN, and RF+ANN models performed worst. It demonstrated that the LASSO, SVM-RFE, and RF models could be used independently to predict the risk of IS.

At present, many IS risk prediction models have been established. In 2021, a case-control study in China developed a LASSO model to better identify IS. The prediction model showed good discrimination, with an AUC of 0.916 for the LASSO method using 14 features (33). In this study, the LASSO, SVM-RFE, and RF models performed well, and the AUC value reached more than 90%. The sensitivity, specificity, and accuracy of LASSO, SVM-RFE, and RF models were still very satisfactory in the test datasets. This indicated that the LASSO, SVM-RFE, and RF diagnostic models have diagnostic robustness and potential utility in detecting IS.

A radiomics study identified the selection of the LASSO combined with the SVM as the optimal method for differentiating gliosarcoma and glioblastoma (34). This result suggested that models constructed by combining several machine learning algorithms may result in better prediction ability than a single algorithm. Therefore, we constructed and validated the LASSO+SVM-RFE and SVM-RFE+RF models of IS. Although the AUC, sensitivity, and specificity of LASSO+SVM-RFE and LASSO+RF models were still very satisfactory, they were still slightly inferior to LASSO, SVM-RFE, and RF models. This result was the opposite of what was expected.

The neural network of deep learning enables the models to scale exponentially with the growing quantity and dimensionality of data, which makes deep learning particularly useful for solving complex problems (35). The growing popularity of deep learning in healthcare has accelerated research into its utility in the complex biology of cancer (36). A study found that ANN is the most suitable diagnostic model based on machine learning in skin cutaneous melanoma (37). In this study, to evaluate the prediction performance of the ANN model, we constructed and validated ANN models for feature genes screened by LASSO, SVM-RFE, and RF, respectively. The sensitivity value, that is, the true positive rate, reached more than 70% in the test dataset. However, the specificity value reached <50% in the test dataset. This showed that the ANN model is efficient at classifying positive samples and is unsuitable at classifying negative samples. This study obtained the predictive ability of each model by constructing and comparing the multiple models of IS, which provided a new method for the early diagnosis and prediction of IS.

This study also had some limitations. First, due to the lack of clinical data on IS in the GEO database, the clinical features of IS were not included in the diagnostic models. In addition, the insufficient sample size of IS in the GEO database may affect the diagnostic effect of the IS model.

## Conclusion

In this study, we constructed and validated the LASSO, SVM-RFE, RF, and ANN disease classification models. The AUC, sensitivity, and specificity indicated that the LASSO, SVM-RFE, and RF models performed well for IS diagnosis and prediction. However, the ANN model is efficient at classifying positive samples and is unsuitable at classifying negative samples. Nevertheless, large-scale and multiple-center studies will be needed to verify our findings.

## Data availability statement

The original contributions presented in the study are included in the article/supplementary material, further inquiries can be directed to the corresponding author/s.

## Author contributions

W-XY and C-GY conceived and designed the study. W-XY, F-FW, and Y-YP made the diagrams and tables of the article. W-XY wrote the manuscript. J-QX and M-HL revised the manuscript. All authors read and approved the final manuscript.

## Funding

This study was supported by the Gansu Province Youth Science and Technology Foundation (21JR7RA421) and the Cuiying Scientific and Technological Innovation Program of Lanzhou University Second Hospital (CY2021-BJ-A16 and CY2020-MS18).

## Conflict of interest

The authors declare that the research was conducted in the absence of any commercial or financial relationships that could be construed as a potential conflict of interest.

## Publisher's note

All claims expressed in this article are solely those of the authors and do not necessarily represent those of their affiliated organizations, or those of the publisher, the editors and the reviewers. Any product that may be evaluated in this article, or claim that may be made by its manufacturer, is not guaranteed or endorsed by the publisher.

## Abbreviations

IS: ischemic stroke
GEO: Gene Expression Omnibus
DEGs: differentially expressed genes
LASSO: least absolute shrinkage and selection operator
SVM-RFE: support vector machine-recursive feature elimination
RF: Random Forest
ANN: artificial neural network
GO: Gene Ontology
BP: biological processes
CC: cellular components
MF: molecular functions
GSEA: gene set enrichment analysis
AUC: area under the curve
ROC: receiver operating characteristic.
